# Randomized double-blind clinical trial comparing safety and efficacy of the biosimilar BCD-022 with reference trastuzumab

**DOI:** 10.1186/s12885-020-07247-9

**Published:** 2020-08-20

**Authors:** Sergey M. Alexeev, Andrey V. Khorinko, Guzel Z. Mukhametshina, Konstantin G. Shelepen, Olga N. Burdaeva, Sergey A. Kulik, Chiradoni Thugappa Satheesh, Kirti Srivastava, Mummaneni Vikranth, Fedor Kryukov, Anastasia N. Paltusova, Mariya S. Shustova, Roman A. Ivanov

**Affiliations:** 1 N.N. Petrov NII of Oncology of the Ministry of Healthcare of the Russian Federation, Moscow, Russian Federation; 2 SBHI of PK Perm Krai, Perm Krai Cancer Dispensary, Perm, Russian Federation; 3 SAHI Republican Clinical Cancer Dispensary of the Ministry of Healthcare of the Republic of Tatarstan, Kazan, Russian Federation; 4 Brest Regional Clinical Cancer Dispensary, Volgograd, Russian Federation; 5 SBHI of Arkhangelsk Region Arkhangelsk Regional Clinical Cancer Dispensary, Arkhangelsk, Russian Federation; 6 KLPU City Cancer Dispensary of the City of Donetsk, Donetsk, DNR Ukraine; 7 Sri Venkateshwara Hospital, Bangalore, India; 8 King Georges Medical University, Lucknow, India; 9 City Cancer Center, Vijayawada, India; 10 JSC BIOCAD, Saint Petersburg, Russian Federation

## Abstract

**Background:**

BCD-022 is a trastuzumab biosimilar which was shown to be equivalent to reference trastuzumab in a wide panel of physicochemical studies as well as preclinical studies in vitro and in vivo. International multicenter phase III clinical trial was conducted to comparatively assess efficacy and safety of BCD-022 and reference trastuzumab in combination with paclitaxel used as the therapy of metastatic HER2(+) breast cancer. Pharmacokinetics and immunogenicity were also studied.

**Methods:**

Patients with no previous treatment for metastatic HER2(+) breast cancer were randomly assigned 1:1 to BCD-022 or reference trastuzumab and were treated with trastuzumab + paclitaxel. Therapy continued for 6 cycles of therapy (every 3 weeks), until progression of the disease or unbearable toxicity. Primary study endpoint was overall response rate. Study goal was to prove equivalent efficacy of BCD-022 and reference trastuzumab. Equivalence margins for 95% CI for difference in overall response rates were set at [− 20%; 20%].

**Results:**

In total 225 patients were enrolled into the study, 115 in BCD-022 arm and 110 in reference trastuzumab arm. Overall response rate was 49.6% in BCD-022 arm and 43.6% in reference trastuzumab arm. Limits of 95% CI for difference of overall response rates between arms were [(− 8.05)-19.89%], thus, they lied within predetermined equivalence margins [− 20%; 20%]. Profile of adverse events was similar between groups (any AEs were reported in 93.81% of patients in BCD-022 arm and 94.55% of patients in reference arm). No unexpected adverse reactions were reported throughout the study. No statistically significant differences regarding antibody occurrence rate (either BAb or NAb) was found between BCD-022 (*n* = 3; 2.65%) and comparator (*n* = 4; 3.64%). Both drug products are characterized with low occurrence rate and short life of anti-trastuzumab antibodies. Pharmacokinetics assessment after 1st and 6th study drug injection also demonstrated equivalent PK parameters by all outcome measures: AUC_0–504_, С_mах_, Т_max_, T_1/2_. Analysis of C_trough_ did not reveal any significant inter-group differences as well.

**Conclusions:**

Thus, results of this study have demonstrated therapeutic equivalence of trastuzumab biosimilar BCD-022 and referent trastuzumab drug.

**Trial registration:**

The trial was registered with ClinicalTrials.gov (Study Number NCT01764022). The date of registration was January 9, 2013.

## Background

### Introduction

Based on GLOBOCAN data there will be an estimated 18.1 million new cancer cases (17.0 million excluding nonmelanoma skin cancer) and 9.6 million cancer deaths (9.5 million excluding nonmelanoma skin cancer) in 2018. Among females, breast cancer is the most commonly diagnosed cancer with over 2 million new cases and the leading cause of cancer death (626,679 deaths per year) [2]. Furthermore, studies have shown that up to 25% of breast cancers have an overexpression of HER2.

Unfortunately, the cost of medicine and the economic conditions of society has limited its access to a small number of patients. Data from the observational studies conducted between the years 2000 and 2015 clearly shows that patients with HER2+ MBC from United States (12%), Europe (27–54%) and China (27.1–49.2%), did not receive Trastuzumab or any other HER2-targeted agent as first and/or later line of treatment [1]. However, the introduction of trastuzumab biosimilars into the market would give access to an alternative yet cheaper therapy to a wider network of patients.

### Objectives

The study was based on the hypothesis of equivalence of BCD-022 (trastuzumab by JSC BIOCAD, Russia) in combination with paclitaxel used as the therapy of inoperable or metastatic HER2(+) breast cancer in comparison with Herceptin® (F. Hoffmann-La Roche Ltd., Switzerland) in combination with paclitaxel. The objectives of the study were to evaluate efficacy, safety and pharmacokinetics of BCD-022 compared with reference trastuzumab by 1. overall response rate and other efficacy parameters; 2. incidence and severity of adverse events; 3. serum concentration after the first and multiple trastuzumab administration; 4. incidence and concentration of anti-trastuzumab antibodies.

## Methods

### Trial design

This Phase III study was approved by independent ethics committees including local independent committees at all participated study sites and performed in accordance with ethical principles set forth in the World Medical Association Declaration of Helsinki “Ethical Principles for Medical Research Involving Human Subjects” or comparable national ethical standards, and International Conference on Harmonization Good Clinical Practice guidelines. All patients provided written informed consent before starting screening procedures. The study was international, multicenter, double-blind, randomized, two-arm, parallel-group trial comparing BCD-022 with reference trastuzumab. The study was conducted on 48 sites in four countries: Russia, Belarus, Ukraine and India from October 2012 to December 2017. The trial was registered with ClinicalTrials.gov (Study Number NCT01764022).

### Participants

The trial included 225 female patients aged 18–75 with metastatic breast cancer characterized by overexpression/amplification of HER2. As per protocol inclusion criteria stated: “Grade 3+ HER2 overexpression confirmed by immunohistochemical (IHC) staining or grade 2+ HER2 overexpression accompanied by HER2 gene amplification confirmed by fluorescent hybridization *in situ* (FISH). Assessments made by a local laboratory are accepted regardless of the time they were performed.” Thus, biopsy materials were not confirmed by an independent laboratory if HER2 status was evaluated and a previous report was available. To be enrolled patient must have had at least one measurable lesion according to RECIST 1.1 on CT scan; ECOG score 0–2; life expectancy of at least 20 weeks. Exclusion criteria encompassed a number of medical conditions, including a history or presence of hypersensitivity; cardiovascular system pathology (CHF stage III-IV according to NYHA classification, unstable angina pectoris, myocardial infarction); uncontrolled hypertension; acute or active chronic infections; unstable CNS metastases or other malignancies, with the exclusion of radically treated basal cell carcinoma of skin or cervical cancer in situ. Previous surgery, radiation therapy, hormonal therapy, use of any experimental medications of non-metastatic breast cancer must have been completed at least 28 days prior randomization. Any previous anticancer therapy for metastatic BC as well as disease progression within 6 months after adjuvant and/or neoadjuvant BC therapy were recognized as exclusion criteria for this trial. Characteristics of the main disease in patients involved in the study (ITT population) by groups are represented in Suppl Table 1.

### Randomization

After completion of 28-days screening period eligible patients were centrally randomized in a 1:1 ratio into 2 treatment arms to receive either BCD-022 or reference trastuzumab. Randomized assignment was stratified according to previous treatment, estrogen and/or progesterone receptor status (expressed/not expressed) and age (< 55/≥55 years).

### Interventions

Patients were treated with BCD-022 or reference trastuzumab at a loading dose of 8 mg/kg (once), followed by maintenance dose of 6 mg/kg every 3 weeks (5 administrations), + paclitaxel 175 mg/m^2^ every 3 weeks as 3-h intravenous infusion (6 administrations). Therapy continued for 6 cycles of therapy (every 3 weeks), until progression of the disease or unbearable toxicity. Therapy were administered as a slow intravenous infusion; infusion speed was corrected according to the scheme provided in reference drug label. Premedication was mandatory before investigational treatment including glucocorticoid (dexamethasone), diphenhydramine (or its equivalent) and cimetidine (or ranitidine). During the trial trastuzumab dose correction was not permitted. Paclitaxel dose adjustment was allowed according to the scheme provided in drug label. After the planned 6 cycles of therapy, patients with complete or partial response or stable disease by the decision of Investigator were transferred to the maintenance therapy period, within which they currently continue receiving unblinded maintenance therapy with trastuzumab (until disease progression or unbearable adverse events). Endocrine therapy was not used in this trial.

1. Such approach in cooncordance with NCCN Guidelines Version 1.2020 Invasive Breast Cancer: Systemic Therapy Regimens For Recurrent Or Stage IV (M1) Disease (HER2-Positive)
2. Trastuzumab main effect is elimination of HER2-positive malignant cells, irrespective of the nature and stage of cancer. Patients with advanced cancers have maximum tumor load, which allows demonstrating the full effect of trastuzumab.
3. No other therapies (e.g. surgery or radiation therapy) are used in the population with metastatic breast cancer, except for those used per BCD-022-2 Protocol. In the absence of additional factors affecting treatment outcome, significance of the analyzed efficacy parameters increases.

### Study procedures

Contrast-enhanced computed tomography for efficacy assessment was performed within 28 days before random assignment (baseline) and then after 3 therapy cycles and after 6 therapy cycles. The tumor response was assessed based on the results of CT scan with contrast and using RECIST 1.1 criteria [3]. In case of primary registration of either complete or partial response, a confirmatory CT scan was made 4 weeks later.

To assess treatment safety on each visit data on adverse events were collected and vital signs were measured (body temperature, heart rate, respiration rate, blood pressure); also, throughout the study complete blood count, blood chemistry, urinalysis, ECG and Echo were controlled.

Blood sampling for immunogenicity assessment was done prior to the first trastuzumab administration (baseline), then 15 ± 1, 64 ± 2, 127 ± 2 and 154 ± 2 days (7 weeks) after the last trastuzumab administration. Presence and concentrations of anti-trastuzumab antibodies were determined centrally using ELISA. Detection of neutralizing anti-trastuzumab antibodies (neutralizing potency of binding antibodies) was performed by validated antiproliferative test in BT-474 cell culture.

Blood sampling for pharmacokinetics analysis were taken on day 1 immediately before start of the 1st trastuzumab infusion, then 1.5, 3 (±15 min), 4.5 (±15 min), 6 (±15 min), 24 ± 1, 96 ± 8, 168 ± 8, 336 ± 8 and 504 ± 8 h (21 days) after first administration immediately before subsequent infusions. Blood samples were collected immediately prior to each trastuzumab administration and in 504 ± 8 h after the 6th drug administration. Additionally, to study pharmacokinetics at steady state, for this purpose blood samples were collected immediately before start of the 6th study or reference drug administration and 1.5, 3 (±15 min), 4.5 (±15 min), 6 (±15 min), 24 ± 1, 96 ± 8, 168 ± 8, 336 ± 8, 504 ± 8 h (21 days) after the 6th trastuzumab infusion.

### Study endpoints

Main study objective was to compare efficacy and safety of BCD-022 and reference trastuzumab. Primary study endpoint was overall response rate (cumulative rate of complete and partial responses) in HER2(+) metastatic breast cancer subjects after receiving up to 6 cycles (18 weeks) of paclitaxel + trastuzumab therapy. Overall response rate is the recommended primary endpoint for clinical trials of biosimilars of anticancer drugs. Treatment responses were assessed by CT scans according to RECIST 1.1 criteria and was centrally evaluated by an independent specialist.

Secondary efficacy endpoints were partial response and complete response rates, rates of stable disease and progressive disease.

Additional secondary endpoints were safety, immunogenicity, pharmacokinetics parameters. Safety endpoints included incidence and types of adverse events, therapy-related adverse events, treatment withdrawal due to adverse events. Immunogenicity endpoints included incidence of antidrug antibody formation and neutralizing activity of detected antibodies. Pharmacokinetics endpoints included area under the serum concentration-time curve (AUC), maximum serum concentration (C_max_), time to maximum serum concentration (T_max_) and trough concentration (C_trough_) of trastuzumab during 6 cycles.

### Statistical analysis

Sample size was calculated using the following variables: 2-sided α = 0.05, study power of 80%. For an equivalence margin (the maximal clinically insignificant difference between the groups) determination historical data were reviewed. Overall response rate (ORR) in metastatic breast cancer patients receiving trastuzumab with paclitaxel at the same doses was 41% compared to 17% in paclitaxel monotherapy group ([6], p. 2). Adding of trastuzumab to standard therapy increases ORR by 24%. According to ICH E10 Guideline the margin generally should not be higher than difference between active control and placebo (or standard therapy) based on past experience in placebo-controlled trials (or active controlled studies) of adequate design under conditions similar to those planned for the new trial [5]. Thus, in current study δ (an equivalence margin) should not be higher 24%. It was hypothesized that 95% CI for the difference between ORR in BCD-022 group and in the reference trastuzumab group will be within the limits of − 20 to 20%, i.e. equivalence criterion δ = 0.2.

Thus, it was needed to enroll 103 patients into each study arm; therefore, with a sample size of 206 patients, the study has 80% power to reject the null hypothesis at α = 0.05.

Efficacy analysis was performed in “modified intention-to-treat” population (mITT, patients who received at least 1 infusion; *n* = 223).

Safety analysis was performed in patients who received at least one dose of either BCD-022 or reference trastuzumab (mITT; *n* = 223).

Analysis of main pharmacokinetic parameters of trastuzumab at first cycle of therapy was performed in patients who received at least one infusion of study therapy and with no more than one missed pharmacokinetics serum sample (*n* = 211). Analysis of main pharmacokinetics parameters of trastuzumab at sixth cycle of therapy was performed in patients who received 6th trastuzumab injection and missed not more than one sample during the 6th therapy cycle (*n* = 69). Trough concentration (C_trough_) of trastuzumab during 6 cycles was analyzed in patients who received all 6 cycles of therapy (6 trastuzumab injections) and missed not more than 1 PK sample before every trastuzumab injection (*n* = 156). Immunogenicity analysis was performed in patients who received at least one infusion of study therapy with at least one post-baseline blood sample for immunogenicity assessment available (*n* = 223).

The primary efficacy outcome measure (ORR after 6 therapy cycles) was analyzed using 95% CI for frequency difference between two arms. The hypothesis of equivalence was accepted if the calculated 95% CI for ORR ratio in groups was within the predefine limits. The primary outcome measure for the efficacy analysis and secondary efficacy outcome measures were compared using exact Fisher test / Yates corrected *x*^2^ test. No covariate corrections were provided in the analysis.

Statistical comparison in pharmacokinetics analysis included main factors (AUC_(0-t),_ C_max_, Т_max_, T_1/2_ and C_through_) and supplementary factors (AUMC, К_el_, MRT, Cl and V_d_). All of them were quantitative data and were presented according to descriptive statistics rules as mean values and SD or as medians and interquartile ranges.

For normally distributed data, it was panned to use two-sample t-test and analysis of variances.

For non-normally distributed data Mann-Whitney test, Wilcoxon test and Kruskal-Wallis test were implemented.

Two or more independent groups were compared by quantitative parameters using ANOVA (one-way analysis of variance), Kruskal-Wallis test and median test.

Processing of categorical data was performed using frequency (one-way) tables, cross (multi-way) tables, exact Fisher test, equality of frequency test, and Pearson’s chi-square test (Yates-corrected test was used for cross tables 2×2). Percentages or proportions were used to describe categorical data.

Feasibility of using different statistical methods was evaluated after completion of the data collection, as the distribution pattern and sample homogeneity were not known in advance.

Statistical analysis was performed using STATISTICA software.

## Results

In total 225 patients with HER2-positive metastatic breast cancer were enrolled into study. Subjects were randomly distributed to treatment groups: 115 were assigned to BCD-022 group and 110 were assigned to reference trastuzumab group. Two randomized patients who have not received at least one study drug administrations were excluded from analysis, as they cannot provide any additional data on the efficacy of study drug. Thus, 223 of 225 randomized subjects were included to mITT population. In general, it is suggested that mITT population is the closest to clinical practice.

**Fig. 1:**
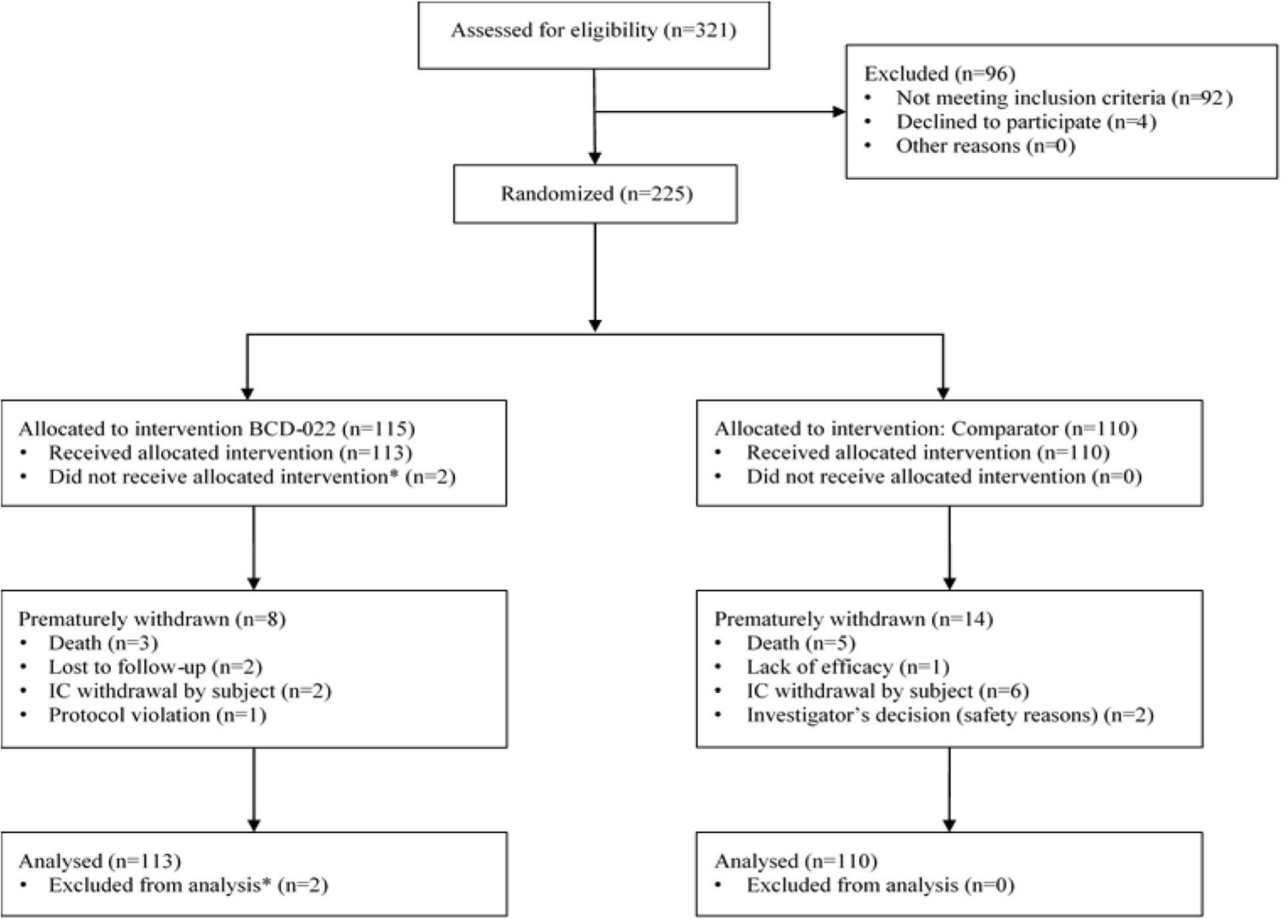
Disposition of patients by study arms and reasons for withdrawal. * Patients was excluded prior to drug administration (1 - IC withdrawal by subject; 1 – death)

Twenty patients were withdrawn prematurely, 8 in BCD-022 arm (3 – death, 2 – IC withdrawal by subject, 2 – lost to follow-up, 1 – protocol violation), 14 in reference trastuzumab arm (5 – death, 6 – IC withdrawal by subject, 1 – lack of efficacy, 2 – physician decision) (Fig. 1).

### Efficacy

According to CT-scan results, the overall response rate (complete and partial response rate), that was the primary outcome measure for the efficacy analysis (main analysis was conducted in mITT population), was 49.6% (95% CI 40.08–59.07) in BCD-022 arm and 43.6% (95% CI 34.31–53.41) in reference trastuzumab arm, respectively (*p* = 0.452, Yates-corrected *x*^2^ test). There were four complete responses in BCD-022 group and two in reference trastuzumab group (*р* = 0.683, exact Fisher test), partial responses were reported in 52 of 113 subjects from BCD-022 arm and in 46 of 110 subjects in reference trastuzumab arm, respectively (*р* = 0.619, Yates-corrected *x*^2^ test) (Table 1).

**Table 1:** Efficacy endpoint assessment results (ITT population)

Parameter	Group 1: BCD-022 (***n*** = 113)		Group 2: Reference Trastuzumab (***n*** = 110)		***p***-value
	N	% (95% CI)	n	% (95% CI)	
**Primary outcome measure**					
ORR	56	49.6 (40.08–59.07)	48	43.6 (34.31–53.41)	0.452^a^
**Secondary outcome measure**					
CR	4	3.5 (1.14–9.35)	2	1.8 (0.32–7.06)	0.683^b^
PR	52	46.0 (36.69–55.62)	46	41.8 (32.6–51.61)	0.619^a^
ST	28	24.8 (17.35–33.95)	21	19.1 (12.46–27.93)	0.388^a^
PD	25	22.1 (15.08–31.10)	28	25.5 (17.84–34.81)	0.670^a^

Note: *ORR* overall response rate, *CR* complete response, *PR* partial response, *ST* stabilization, *PD* progressive disease, *NER* non-evaluable response

^a^ Pearson’s χ^2^-test with Yates correction, ^b^ exact Fisher’s test

The primary outcome measure for the efficacy analysis in mITT study population was analyzed using 95% CI for the difference in response rates between two groups. The difference in overall response rate between BCD-022 and reference trastuzumab arms counted 6.0%, the result of 95% CI calculation for the differences in overall response rate between two compared groups was [(− 8.05)-19.89%]. The confidence interval lies within the predefined range of clinically insignificant difference, so conclusion on equivalent efficacy of BCD-022 and reference trastuzumab was made. The equivalence of efficacy confirms biosimilarity of BCD-022 and reference trastuzumab.

**Fig. 2:**
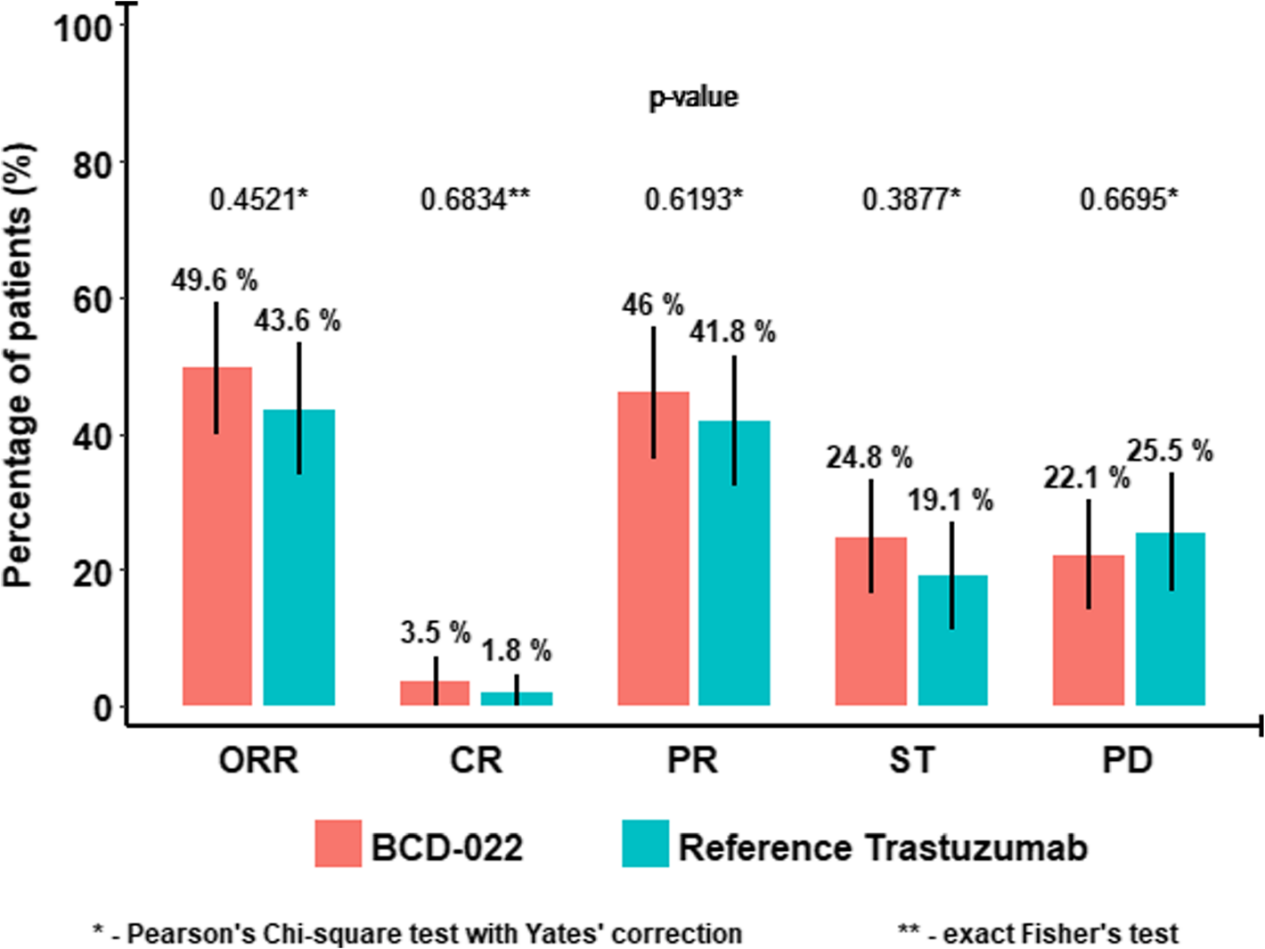
Efficacy endpoint assessment results (mITT population). *-Pearson's Chi-square test with Yates' correction. **-exact Fisher's test

Comparison of other efficacy assessment parameters (secondary outcome measures) did not reveal any statistically significant differences between study arms in mITT population (Fig. 2).

Additional analysis of efficacy in PP population confirmed the results of analysis in mITT population. According to CT-scan results, the overall response rate (complete and partial response rate) in PP population was 51.4% (95% CI 41.66–60.99) in BCD-022 arm and 49.5% (95% CI 39.25–59.76) in reference trastuzumab arm, respectively (*p* = 0.895, Yates-corrected *x*^2^ test). The difference in overall response rate between BCD-022 and reference trastuzumab arms counted 1.9, 95% CI for difference of overall response rate was [(− 12.76)-16.54%]. The limits of confidence interval lie within predefined range of clinically insignificant differences, so conclusion on biosimilarity of BCD-022 and reference trastuzumab is confirmed in PP population.

### Safety

Overall, some adverse events were reported in 106 (93.81%) patients from BCD-022 arm and in 104 (94.55%) patients from the reference trastuzumab arm. Most AEs were associated by the main disease or myelosuppressive chemotherapy (paclitaxel).

One treatment discontinuation (followed by withdrawals) due to AEs/SAEs were reported in comparator arm. For this patient following SAEs were registered: neutropenia grade 4, hepatic veins compression grade 4.

Study arms had no significant difference regarding the occurrence rate of any SAEs as well as no differences in occurrence rate of SAEs related to the study therapy (*р* > 0.05). SAE were revealed in 21 patients: 8 (7.08%) patients from BCD-022 group and 13 (11.82%) patients from the comparator group (*p* = 0.327). According to investigators, there were 9 (4.04%) SAEs related to the study therapy: 4 (3.54%) SAEs in BCD-022 and 5 (4.55%) SAEs in comparator group. Generally, SAEs were associated with the underlying pathology, chemotherapy agents used in combination therapy or with other factors unrelated to the study therapy.

In total, during the study, 8 lethal outcomes were reported: 3 (2.65%) patient from BCD-022 group and 5 (4.55%) patient from the comparator group, with no significant difference revealed (*р* = 0.495). Two lethal outcomes (one in each arm) were registered as probably related to the study drug. (Table 2).

**Table 2:** Safety assessment results by primary and secondary outcome measures

Parameter	Group				***p***-value
	Group 1: BCD-022 (***n*** = 113)		Group 2: Reference Trastuzumab (***n*** = 110)		
	n	%	n	%	
Any AE (including SAE)	106	93.81	104	94.55	1.000^a^
SAE	8	7.08	13	11.82	0.326^a^
Therapy-related SAE	4	3.54	5	4.55	0.746^b^
Courses postponed due to AE/SAE	4	3.54	5	4.55	0.746^b^
Courses discontinued due to AE/SAE	0	0.00	1	0.91	0.493^b^
Lethal outcomes	3	2.65	5	4.55	0.495^b^

Note: This tabulation does not include the lethal outcome in patient who was randomized but did not receive a single dose of the study drug;

^a^Yates-corrected χ^2^ test; ^b^two-tailed Fisher exact test

In general, throughout the study a total incidence of grade 3–5 adverse events was 189 cases for both arms, 94 (49.74%) and 95 (50.26%) cases were registered in BCD-022 and reference trastuzumab groups respectively (*p* = 1.0). Grade 3–5 adverse events (CTCAE v4.03) were mostly reported for hematology disorders such as neutropenia, leukopenia, lymphocytopenia and anemia. These AEs are expected in breast cancer subjects undergoing myelosuppressive chemotherapy with paclitaxel.

No statistically significant differences were found between study arms in frequencies of abnormalities in all assessed vital signs laboratory values.

During the analysis no significant difference between study arms were found by any of the adverse events. Data shown above clearly demonstrated that safety profile of BCD-022 was not significantly different from that of reference drug. Moreover, safety profile in both arms was consistent with literature data on reference trastuzumab safety.

### Immunogenicity

As per protocol, the screening test was performed to reveal the presence of binding antibodies (BAb) in subject’s blood, followed by the confirmatory analysis. If binding antibodies were found, the test for neutralizing antibodies (NAb) was performed. Upon assessment of binding antibodies, the neutralizing potency of anti-trastuzumab antibodies in patients’ samples was evaluated. Neutralizing activity was revealed in 3 (2.65%) patients from the study arm and 4 (3.64%) patients from the comparator arm (*р* = 1.000, two-tailed Fisher exact test).

Thus, no statistically significant differences regarding antibody occurrence rate (either BAb or NAb) was found between BCD-022 and reference trastuzumab. Both drug products are characterized with low occurrence rate and short life of anti-trastuzumab antibodies.

### Pharmacokinetics

The analysis has shown that upon both single-dose and repeated-dose administration of study drug and comparator, the changes in serum concentrations of trastuzumab were similar.

After a single administration (1 cycle) of the study drug or the comparator, AUC_(0–504)_ was 28,969,372.5 [20,165,337.0; 36,096,712.5] (ng/ml)·h for BCD-022 and 28,796,527.9 [20,430,685.5; 36,232,918.5] (ng/ml)·h for reference trastuzumab (*p* > 0.05). Maximum serum concentrations of trastuzumab (C_max_) after the administration of BCD-022 and comparator were 218,720.0 [178,270.0; 264,700.0] and 216,710.0 [186,740.0; 269,780.0] ng/ml, respectively. 90% confidence interval for the ratios of AUC_(0–504)_ in BCD-022 and reference trastuzumab groups was [87.66, 109.01%], and for C_max_ — [90.89, 106.03%]. Both calculated confidence intervals lie in within acceptable range (80–125%), thus the PK of study drugs is considered to be equivalent.

After a 6th administration (6 cycle) of the study drug or the comparator, AUC_(0–504)_ was 25,800,936.8 [21,150,486.0; 33,066,277.5] (ng/ml)·h for BCD-022 and 26,730,362.3 [22,137,053.3; 31,240,387.5] (ng/ml)·h for reference trastuzumab (*p* > 0.05). Maximum serum concentrations of trastuzumab (C_max_) after the 6th administration of BCD-022 and comparator were 168,735.0 [148,810.0; 204,870.0] and 169,220.0 [150,140.0; 179,230.0] ng/ml, respectively. 90% confidence interval for the ratios of AUC_(0–504)_ in BCD-022 and reference trastuzumab groups was [89.65, 123.27%], and for C_max_ — [94.01, 116.18%]. Both calculated confidence intervals lie in within acceptable range (80–125%), thus the PK of study drugs is considered to be equivalent.

Calculated median C_trough_ (for 6 sampling points prior to each administration) was 19,380.0 ng/ml in ВCD-022 arm and 21,572.5 ng/ml in reference trastuzumab arm, no difference was revealed between the study arms with respect to this parameter (*р* = 0.210, two-tailed Mann-Whitney test).

According to the results of the study the equivalence of PK profiles of BCD-022 and reference trastuzumab is confirmed.

Thus, the absence of any differences between the study arms with respect to all mentioned above parameters confirms that pharmacokinetics of BCD-022 is equivalent to that of reference trastuzumab.

## Discussion

The development of biosimilar products is a complex, step-by-step process which involves factoring in a wide range of parameters such as physicochemical properties, functional characteristics, efficacy and safety. Even though regulatory guidelines are in place for such products, they lack the specifications and guidelines to use it in a clinical setting. As stated by Nixon et al. [7], the use of biosimilar products is not a straightforward process as it needs to consider several stakeholders. However, this limitation does create a control over the costs of cancer treatment.

The study drug BCD-022 was registered in the Russian Federation under the name Herticad® in December 2015 and has been in use in the Russian clinical practice since March 2016. The initial results published by Kolyadina et al. [8] highlights the effectiveness, safety and economic rationality of Herticad® in neoadjuvant chemotherapy in HER2+ breast cancer. Moreover, it showed the obvious cost benefit of cancer therapy with a decrease in costs by 75% from March 2016 to December 2017.

Similarities between the efficacy and safety data between Herticad® and the reference product, coupled with a 66% lower price in the Russian market for Herticad®, has enabled patients to have broader access to vital therapy and in turn, save additional costs in the healthcare sector. After the market entry of Herticad® the average annual treatment cost per patient with trastuzumab fell by 62%. The number of eligible patients who gained access to the treatment with trastuzumab increased from 41% in 2015 to 56% in 2017 [4] Market entry of the biosimilar drugs to state segment in Russia. Pharmacoeconomic and social impact for the respective INNs). According to the Headway Monitoring data on Russian state procurements and BIOCAD unpublished data, in 2019 in forty-seven regions of Russian Federation 100% of patients requiring trastuzumab therapy receive it and in thirty regions more the access to trastuzumab is more than 50%.

Although Herticad® was investigated only in metastatic breast cancer it has been approved for early breast cancer, metastatic breast cancer and metastatic gastric cancer as well as the innovator trastuzumab. In Europe, it is a common practice to extrapolate biosimilar agents to indications for which it was not tested during the clinical trial. According to ЕМА «Guideline on similar biological medicinal products containing monoclonal antibodies – non-clinical and clinical issues»: *«Extrapolation of clinical efficacy and safety data to other indications of the reference mAb, not specifically studied during the clinical development of the biosimilar mAb, is possible based on the overall evidence of comparability provided from the comparability exercise and with adequate justification”.* As for Trastuzumab biosimilar; the active substance interacts with one active site in HER2 receptors and has no different impact in the tested and non-tested therapeutic indications. In the trial BCD-022-02, the therapeutic indication is relevant in terms of efficacy and safety and the homogeneous representative population with metastatic breast cancer and HER2 overexpression, and/or amplification is sensitive for differences in all relevant aspects of efficacy and safety. As it was stated above, BCD-022 (Herticad®, JSC BIOCAD, Russia) and reference Trastuzumab (Herceptin®, F.Hoffmann-La Roche, Switzerland) are comparable regarding efficacy, safety and pharmacokinetic profiles when used in combination with paclitaxel in mBC HER2+ patients. Therefore, no additional data is required for efficacy and safety extrapolation of BCD-022 (Herticad®, JSC BIOCAD, Russia) to all indications.

With the number of biosimilars increasing, more marketed medicines are expected to reach the market over the next few years and will certainly provide a cost-effective treatment to a greater number of patients. Among the different clinical applications of biosimilar medicines, cancer treatment remains the main target area. Usage data for Trastuzumab biosimilar (Herticad®, JSC BIOCAD, Russia) in routine clinical practice for patients with HER2-positive breast cancer is currently being collected. Thus, a comprehensive pharmacovigilance study is ongoing, and the marketed biosimilar product is being constantly monitored; providing more useful information to clinicians regarding the safety and efficacy of this medicine.

## Conclusions

Results of international multicenter phase III clinical trial have demonstrated therapeutic equivalence of trastuzumab biosimilar BCD-022 and referent trastuzumab drug (NCT01764022). As it was stated above, BCD-022 (Herticad®, JSC BIOCAD, Russia) and reference Trastuzumab (Herceptin®, F.Hoffmann-La Roche, Switzerland) are comparable regarding efficacy, safety and pharmacokinetic profiles when used in combination with paclitaxel in patients with metastatic breast cancer and HER2 overexpression. This has eliminated the need to provide additional data to extrapolate the efficacy and safety of BCD-022 (Herticad®, JSC BIOCAD, Russia) against all indications. Unfortunately, the economic conditions of the society and the cost of the drug has limited its accessibility to a small proportion of patients. Therefore, the introduction of Trastuzumab biosimilars into the market has provided a wider access to an alternative yet cheaper therapy to a broader network of patients.

## Supplementary information

**Additional file 1: Supplemental Table 1.** Characteristics of the main disease in patients involved in the study (ITT population) by groups.

## Data Availability

The datasets generated and/or analyzed during the current study are not publicly available due to containing information that could compromise research participants privacy and consent under Russian Federal Law No. 323-FZ and Russian Federal Law No. 61-FZ, but are available from the corresponding author on reasonable request with the consent of the participants.

## Abbreviations

AE: Adverse Event
ANOVA: One-way analysis of variance
AUC: Area Under Curve
AUMC: Area Under The First Moment Curve
BAb: Binding Antibodies
BC: Breast Cancer
CHF: Congestive Heart Failure
CI: Confidence Interval
Cl: Clearance
C_max_: Peak Serum Concentration
CNS: Central Nervous System
CR: Complete Response
CT: Computer Tomography
CTCAE: Common Terminology Criteria for Adverse Events
ECG: Electrocardiography
ECOG: Eastern Cooperative Oncology Group
ELISA: Enzyme-linked Immunosorbent Assay
EMA: European Medicines Agency
FISH: Fluorescent in situ Hybridization
HHS: Health and Human Services
IC: Informed Consent
IHC: Immunohistochemistry
INN: International Nonproprietary Name
ITT: Intention-To-Treat
IV: Intravenous
JSC: Joint Stock Company
К_el_: Elimination Rate Constant
MBC: Metastatic Breast Cancer
mITT: Modified Intention-To-Treat
MRT: Mean Residence Time
NAb: Neutralizing Antibodies
NCCN: National Comprehensive Cancer Network
NER: Non-Evaluable Response
NYHA: New York Heart Association
ORR: Overall Response Rate
PD: Progressive Disease
PK: Pharmacokinetics
PP: Per Protocol
PR: Partial Response
RECIST: Response Evaluation Criteria In Solid Tumors
SAE: Serious Adverse Event
SD: Standard Deviation
ST: Stabilization
T_1/2-_: Half-life Period
T_max_: Time To Reach Peak Serum Concentration
V_d_: Volume of Distribution
