# Supplementary material for: Randomized double-blind clinical trial comparing safety and efficacy of the biosimilar BCD-022 with reference trastuzumab
Source: BMC Cancer. 2020 Aug 20;20:783. doi: 10.1186/s12885-020-07247-9 (PMC7439710; doi:10.1186/s12885-020-07247-9)
Supplement: Supplementary file 1 — Additional file 1: Supplemental Table 1. Characteristics of the main disease in patients involved in the study (ITT population) by groups. [file 12885_2020_7247_MOESM1_ESM.docx]

**Supplemental Table 1:** Characteristics of the main disease in patients involved in the study (ITT population) by groups

| **Parameter** | **Group** | | | | **p-value** |
| --- | --- | --- | --- | --- | --- |
|  | **Group 1:**  **BCD-022**  **(n = 113)** | | **Group 2:**  **Herceptin^®^**  **(n = 110)** | |  |
|  | **n** | **%** | **N** | **%** |  |
| **HER-2 expression** | | | | | |
| 3+. n (%) | 98 | 86.96 | 97 | 88.18 | 1.000* |
| 2+. n (%) | 14 | 12.17 | 13 | 11.82 |  |
| No data | 1 | 0.88 | 0 | 0.00 |  |
| **Hormone receptors expression** | | | | | |
| Estrogen receptors | 39 | 34.78 | 38 | 34.55 | 0.952* |
| Progesterone receptors | 13 | 11.30 | 11 | 10.00 |  |
| No expression | 60 | 53.04 | 61 | 55.45 |  |
| No data | 1 | 0.88 | 0 | 0.00 |  |
| **Morphology** | | | | | |
| Ductal | 36 | 31.86 | 33 | 30.00 | 0.908* |
| Medullar | 3 | 2.65 | 4 | 3.65 |  |
| Lobular | 23 | 20.35 | 18 | 16.36 |  |
| Tubular | 33 | 29.20 | 37 | 33.63 |  |
| Mucinous | 3 | 2.65 | 5 | 4.54 |  |
| Micropapillary | 1 | 0.88 | 0 | 0.00 |  |
| Unclassified | 14 | 12.39 | 13 | 11.82 |  |
| **Prior therapy for breast cancer** | | | | | |
| Any prior therapy | 61 | 53.91 | 62 | 56.36 | 0.824** |
| No prior therapy | 52 | 46.09 | 48 | 43.64 |  |
| **Type of chemotherapy** | | | | | |
| Neoadjuvant chemotherapy | 17 | 15.04 | 10 | 9.09 | 0.247** |
| Adjuvant chemotherapy | 37 | 32.74 | 41 | 37.27 | 0.570** |
| **Type of radiotherapy** | | | | | |
| Neoadjuvant radiotherapy | 9 | 7.96 | 5 | 4.55 | 0.409* |
| Adjuvant radiotherapy | 29 | 25.66 | 31 | 28.18 | 0.785** |
| Palliative radiotherapy | 4 | 3.54 | 4 | 3.64 | 1.000* |
| **Disease duration, months** | | | | | |
| Mean | 23.53 | | 20.96 | | 0.545*** |
| Median | 15.80 | | 14.29 | |  |
| Minimum | 0.26 | | 0.03 | |  |
| Maximum | 150.08 | | 103.29 | |  |
| Lower quartile | 5.26 | | 2.79 | |  |
| Upper quartile | 31.28 | | 27.40 | |  |
| SD | 27.58 | | 22.85 | |  |
| CV % | 117.22 | | 109.05 | |  |
| Note: *two-tailed Fisher exact test; **$\chi^{2}$ test; ***two-tailed Mann-Whitney test | | | | | |
